# Multiomics Biomarkers for Differential Diagnosis of Pleural Effusion: Integration of Proteomic Markers and Single‐Cell Transcriptomics

**DOI:** 10.1155/humu/5946608

**Published:** 2026-04-15

**Authors:** Zhengyou Zhang, Shaowei Zhan, Ying Tang, Zhougui Ling, Jinyan Wu, Dong Rui, Xiaomou Wei, Moyu Ming

**Affiliations:** ^1^ Department of Pulmonary and Critical Care Medicine, The Fourth Affiliated Hospital of Guangxi Medical University, Liuzhou, China, gxmu.edu.cn; ^2^ Clinical Science Research Center, The Fourth Affiliated Hospital of Guangxi Medical University, Liuzhou, China, gxmu.edu.cn

**Keywords:** adenosine deaminase, biomarkers, driver mutations, EGFR, immune profiling, pleural effusion, single-cell RNA sequencing, targeted therapy, tumor markers

## Abstract

**Background:**

Differential diagnosis of pleural effusion remains challenging despite medical thoracoscopy (MT). We investigated whether integrating proteomic biomarkers with single‐cell transcriptomics and genomic mutation profiling could improve diagnostic accuracy and reveal mechanistic insights.

**Methods:**

We prospectively enrolled 564 patients with pleural effusion undergoing medical thoracoscopy. Pleural fluid biomarkers (adenosine deaminase, carcinoembryonic antigen, cytokeratin‐19 fragment, neuron‐specific enolase) were measured. Single‐cell RNA sequencing profiled immune landscapes across disease etiologies. Driver mutation profiling was performed on malignant pleural effusion samples using targeted next‐generation sequencing encompassing 15 cancer‐related genes. Diagnostic performance was evaluated against histopathological diagnosis.

**Results:**

Final diagnoses included inflammatory PE (*n* = 95, 16.8%), tuberculous PE (*n* = 299, 53.0%), and malignant PE (*n* = 170, 30.1%). For tuberculous PE, ADA achieved AUC 0.916 (sensitivity 83.3% and specificity 89.4%). For malignant PE, combined CEA/CYFRA21‐1 achieved AUC 0.957 (sensitivity 98.2% and specificity 98.7%). Single‐cell analysis revealed distinct immune signatures: Tuberculous PE showed M1 macrophage polarization (M1/M2 ratio 9.48) strongly correlating with ADA levels (rho = 0.68, *p* < 0.001), whereas malignant PE exhibited immunosuppressive features with elevated M2 macrophages and reduced NK cells. Mutation profiling of malignant PE revealed EGFR (46.5%), TP53 (34.7%), and PIK3CA (8.2%) as the most frequently mutated genes. EGFR‐mutant tumors exhibited significantly higher CEA levels (*p* = 0.033) and more immunosuppressive microenvironments with increased M2 macrophages (*p* < 0.001) and decreased CD8+ T cells (*p* < 0.001). The sequential multibiomarker algorithm achieved 96.5% sensitivity and 98.7% specificity for malignant PE detection, with 85.3% overall three‐way classification accuracy.

**Conclusions:**

Multiomics integration combining proteomic biomarkers with single‐cell immune profiling and genomic mutation characterization achieves high diagnostic accuracy for pleural effusion while revealing disease‐specific immune mechanisms and mutation‐driven therapeutic opportunities.

## 1. Introduction

Pleural effusion affects approximately 1.5 million patients annually in the United States, with differential diagnosis among inflammatory, tuberculous, and malignant etiologies remaining a clinical challenge [1, 2]. Medical thoracoscopy provides direct visualization and tissue sampling but is invasive, requires specialized facilities, and carries procedural risks including pain, bleeding, and infection [3, 4]. Development of less invasive diagnostic approaches with comparable accuracy remains a priority [5, 6].

Biomarker‐based approaches offer potential advantages of being less invasive, more accessible, and providing rapid results. Adenosine deaminase (ADA) has been extensively studied for tuberculous pleural effusion diagnosis [7, 8], whereas tumor markers including carcinoembryonic antigen (CEA) and cytokeratin‐19 fragment have shown variable sensitivity for malignant pleural effusion (MPE) [9, 10]. However, individual biomarkers often lack sufficient diagnostic accuracy, and optimal combination strategies remain inadequately defined [11, 12].

Recent advances in single‐cell transcriptomics enable high‐resolution immune profiling, revealing cellular composition and transcriptional states at unprecedented detail. Single‐cell RNA sequencing has uncovered disease‐specific immune signatures in various malignancies and inflammatory conditions [13], but its application to pleural effusion differential diagnosis remains limited [14]. Furthermore, the genomic mutation landscape of MPE, particularly driver mutations in genes such as EGFR, TP53, and KRAS, has been shown to influence both tumor biology and the immune microenvironment [15]. Understanding mutation–immune interactions in the pleural space could inform both diagnostic strategies and therapeutic decision‐making, especially regarding targeted therapy and immunotherapy selection.

We hypothesized that combining optimized multibiomarker panels with single‐cell immune landscape characterization and genomic mutation profiling would achieve diagnostic performance comparable with invasive reference standards while providing mechanistic understanding of disease pathophysiology and identifying actionable therapeutic targets.

## 2. Methods

### 2.1. Study Design and Participants

This prospective cohort study enrolled consecutive adult patients with undiagnosed moderate‐to‐large pleural effusion at a tertiary referral center between January 2020 and December 2022 (Figure 1a). Inclusion criteria were as follows: age 18 years or older, undiagnosed pleural effusion requiring diagnostic workup, and ability to provide informed consent. Exclusion criteria included known diagnosis prior to referral, contraindication to thoracentesis or thoracoscopy, pregnancy, and inability to provide informed consent. The study protocol was approved by the institutional review board (Approval Number: IRB‐2020‐0145), and written informed consent was obtained from all participants.

### 2.2. Sample Collection and Processing

Pleural fluid samples (minimum 10 mL) were collected during initial thoracentesis using sterile technique with an 18‐gauge thoracentesis needle (Cook Medical, Bloomington, Indiana, United States; catalog #G16785). Samples were immediately transferred to BD Vacutainer EDTA tubes (BD Biosciences, Franklin Lakes, New Jersey, United States; catalog #367863) and centrifuged at 1500 × g for 10 min at 4°C using an Eppendorf 5810R refrigerated centrifuge (Eppendorf AG, Hamburg, Germany). Supernatants were aliquoted into 1.5 mL cryogenic vials (Thermo Fisher Scientific, Waltham, Massachusettes, United States; catalog #5000‐1020) and stored at −80°C in a Thermo Forma 906 Ultra‐Low Temperature Freezer (Thermo Fisher Scientific) until analysis. Freeze‐thaw cycles were strictly avoided.

### 2.3. Biomarker Measurements

ADA activity was measured using ADA Assay Kit (Diazyme Laboratories, Poway, California, United States; catalog #DZ117A‐K) based on colorimetric enzymatic method with Berthelot reaction. The assay measures ammonia production from adenosine deamination with sensitivity of 0.5 U/L and linear range 0–150 U/L. Intra‐assay coefficient of variation (CV) was 2.8% and interassay CV was 4.2%. CEA, cytokeratin‐19 fragment (CYFRA21‐1), and neuron‐specific enolase (NSE) were measured using electrochemiluminescence immunoassay (ECLIA) on Cobas e 601 analyzer (Roche Diagnostics, Basel, Switzerland). CEA Elecsys assay (catalog #11731629322, measuring range 0.2–1000 ng/mL, intra‐assay CV 1.8%, interassay CV 3.5%), CYFRA21‐1 Elecsys assay (catalog #12133865122, measuring range 0.1–1000 ng/mL, intra‐assay CV 2.2%, interassay CV 4.1%), and NSE Elecsys assay (catalog #11929216322, measuring range 0.05–370 ng/mL, intra‐assay CV 2.0%, interassay CV 3.8%) were performed according to manufacturer protocols. All assays were performed in a CLIA‐certified laboratory by technicians blinded to clinical diagnoses. Quality control included daily two‐level calibration using PreciControl Tumor Marker controls (Roche Diagnostics, catalog #12172143122) and participation in external quality assessment schemes (College of American Pathologists).

### 2.4. Single‐Cell RNA Sequencing Data and Analysis

To achieve comprehensive immune profiling, we utilized publicly available single‐cell RNA sequencing data from the Gene Expression Omnibus (GEO) database (accession: GSE185058), originally generated by Huang et al. [14]. This dataset comprises 62,382 cells from MPE and matched peripheral blood samples of 5 NSCLC patients, sequenced on the 10X Genomics Chromium platform (10X Genomics, Pleasanton, California, United States) using Chromium Single Cell 3 ^′^ Library & Gel Bead Kit v3. Libraries were sequenced on Illumina NovaSeq 6000 platform (Illumina Inc., San Diego, California, United States). To enable disease‐stratified immune profiling across inflammatory, tuberculous, and MPE etiologies, we complemented the GSE185058 MPE data with computational immune deconvolution of our 564‐patient cohort using CIBERSORTx [16]. Specifically, a signature matrix was constructed from the GSE185058 single‐cell reference to define immune cell type expression profiles. This signature matrix was then applied to bulk RNA expression profiles estimated from pleural fluid cell pellet samples in our cohort to infer immune cell fractions for each disease group. The disease‐stratified immune cell proportions (Figure 2c) and associated biomarker‐immune correlation analyses were derived from this CIBERSORTx deconvolution approach, whereas the UMAP visualization (Figure 2a) and cell–type‐specific gene expression analyses (Figure 2d,e) were derived directly from the GSE185058 scRNA‐seq reference data. Raw count matrices from GSE185058 underwent quality control using Seurat v4.0.6 (R package, CRAN) with cells filtered for 200–5000 genes detected and < 20% mitochondrial gene content. After filtering, high‐quality cells remained for downstream analysis. Data normalization was performed using LogNormalize method with scale factor 10,000. Highly variable genes (*n* = 2000) were identified using variance‐stabilizing transformation (vst method). Principal component analysis was performed on scaled data with Top 30 principal components retained. UMAP dimensionality reduction was computed using uwot v0.1.14 (R package) with default parameters (n_neighbors = 30, min_dist = 0.3). Graph‐based clustering using Louvain algorithm (resolution = 0.5) implemented in Seurat identified 10 immune populations. Cell types were annotated using canonical markers: CD4+ T cells (CD3D + CD4 + CD8A−), CD8+ T cells (CD3D + CD8A + CD8B+), B cells (MS4A1 + CD79A+), monocytes (CD14 + LYZ + S100A8+), M1 macrophages (CD68 + FCGR3A + IL1B + TNF+), M2 macrophages (CD68 + CD163 + MRC1 + CD206+), NK cells (NKG7 + GNLY + NCAM1+), dendritic cells (FCER1A + CD1C + CLEC9A+), plasma cells (SDC1 + MZB1 + CD38high), and neutrophils (FCGR3B + CSF3R + MPO+). Cell type annotations were validated using SingleR v1.10.0 (Bioconductor package) with Human Primary Cell Atlas reference dataset and showed 94.3% concordance with manual annotations. We acknowledge that the GSE185058 dataset originates from MPE patients only; the disease‐stratified immune comparisons rely on CIBERSORTx deconvolution of our cohort samples rather than direct single‐cell profiling of all three PE etiologies. Direct pleural fluid scRNA‐seq across inflammatory, tuberculous, and malignant PE should be pursued in future studies for definitive validation.

### 2.5. Driver Mutation Profiling of MPE

To characterize the genomic mutation landscape of MPE and explore mutation–immune–biomarker interactions, we performed targeted next‐generation sequencing (NGS) on cell‐free DNA extracted from pleural fluid supernatant of all 170 malignant PE patients. DNA was extracted using QIAamp Circulating Nucleic Acid Kit (Qiagen, Hilden, Germany; catalog #55114) according to manufacturer protocol. Library preparation was performed using the xGen Prism DNA Library Prep Kit (Integrated DNA Technologies, Coralville, Iowa, United States) with a custom capture panel targeting 15 cancer‐related genes frequently mutated in nonsmall cell lung cancer: EGFR, TP53, KRAS, ALK, ROS1, BRAF, MET, HER2 (ERBB2), RET, PIK3CA, PTEN, STK11, KEAP1, NF1, and CDKN2A. Sequencing was performed on Illumina NextSeq 550 platform (Illumina Inc., San Diego, California, United States) with target coverage depth of 1000×. Variant calling was performed using GATK Mutect2 (v4.2.6.1) with matched normal filtering. Variants with allele frequency ≥ 1% and minimum supporting reads ≥ 5 were retained. Annotation was performed using ANNOVAR with ClinVar, COSMIC, and OncoKB databases. Mutation frequencies were compared against publicly available TCGA‐LUAD data (*n* = 567) and published MPE cohorts from East Asian populations. Co‐occurrence and mutual exclusivity analyses were performed using Fisher exact test with Benjamini–Hochberg correction. Mutation‐biomarker and mutation‐immune associations were assessed using Wilcoxon rank‐sum test and Spearman correlation.

### 2.6. Medical Thoracoscopy and Histopathological Analysis

All patients underwent medical thoracoscopy using LTF‐240 semirigid thoracoscope with 7 mm outer diameter (Olympus Corporation, Tokyo, Japan) under local anesthesia (lidocaine hydrochloride 2%, AstraZeneca, Cambridge, United Kingdom) with conscious sedation (midazolam 2–5 mg IV, Roche, Basel, Switzerland; fentanyl 50–100 mcg IV, Janssen Pharmaceuticals, Beerse, Belgium) following British Thoracic Society guidelines [3]. The procedure was performed through single port entry with 10 mm trocar (Karl Storz, Tuttlingen, Germany; catalog #27305AA). Multiple pleural biopsies (≥ 6 specimens, each 2–4 mm diameter) were obtained from visually abnormal areas using rigid biopsy forceps (Karl Storz; catalog #65321FD). Specimens were immediately fixed in 10% neutral buffered formalin (Sigma‐Aldrich, St. Louis, Missouri, United States; catalog #HT501128) for 24 h, then processed for paraffin embedding. Sections (4 mcm thickness) were cut and stained with hematoxylin and eosin (H&E). Immunohistochemistry was performed using BenchMark ULTRA automated staining platform (Ventana Medical Systems, Tucson, Arizona, United States). Final diagnoses were adjudicated by multidisciplinary team consensus integrating medical thoracoscopy findings, histopathology, microbiology, clinical course, and minimum 6‐month clinical follow‐up. Inflammatory pleural effusion was diagnosed when inflammation was present without specific infectious or malignant etiology and resolved with conservative management. Tuberculous pleural effusion diagnosis required positive Mycobacterium tuberculosis culture, granulomatous inflammation on histology, or clinical diagnosis with ADA > 40 U/L combined with response to antituberculosis therapy per WHO criteria [7, 8]. MPE required cytological or histological confirmation of malignancy with definitive tumor cell identification.

### 2.7. Statistical Analysis

Sample size calculation was performed using pROC package v1.18.4 (R‐CRAN) with alpha = 0.05, power = 0.90, assuming AUC difference of 0.10, yielding minimum requirement of 520 patients. Continuous variables are presented as median with interquartile range (IQR) or mean with standard deviation (SD) depending on normality assessed by Shapiro–Wilk test. Categorical variables are presented as frequencies and percentages. Between‐group comparisons for continuous variables used Kruskal–Wallis test with Dunn′s post hoc multiple comparison for nonnormally distributed data. Receiver operating characteristic (ROC) curve analysis was performed using pROC package v1.18.4 with 2000 stratified bootstrap replicates for 95% confidence interval estimation. Area under the curve (AUC) values were calculated by DeLong′s method. Optimal cutoff values were determined by Youden′s index. Multivariable logistic regression was performed using glm function. Single‐cell differential gene expression analysis was performed using FindMarkers function in Seurat v4.0.6 implementing Wilcoxon rank‐sum test with Benjamini–Hochberg false discovery rate (FDR) correction. Spearman rank correlation coefficients were calculated to examine associations between biomarker levels and immune cell proportions. To validate the diagnostic algorithm, the cohort was partitioned into a training set (*n* = 376, 66.7%) and a validation set (*n* = 188, 33.3%) using stratified random sampling. All statistical analyses were performed using R version 4.3.1 (R Foundation for Statistical Computing, Vienna, Austria). Two‐sided *p* values < 0.05 were considered statistically significant for all tests.

## 3. Results

### 3.1. Cohort Characteristics

Study cohort and patient characteristics between January 2020 and December 2022, 650 patients with undiagnosed pleural effusion were screened and 564 enrolled after excluding 86 patients (incomplete data *n* = 52, lost to follow‐up *n* = 34) (Figure 1a). Final diagnoses included inflammatory pleural effusion in 95 patients (16.8%), tuberculous pleural effusion in 299 patients (53.0%), and MPE in 170 patients (30.1%) (Figure 1a, Table S1). The cohort comprised 374 males (66.3%) with mean age 56.1 ± 17.2 years. Medical thoracoscopy was successful in all patients, with histopathological confirmation achieved in 510 patients (90.4%). The remaining 54 patients (9.6%) were diagnosed through multidisciplinary team consensus integrating MT visual findings, microbiological results (including positive mycobacterial culture in 38 tuberculous cases), clinical diagnosis based on ADA > 40 U/L with response to antituberculosis therapy per WHO criteria, and minimum 6‐month clinical follow‐up confirming diagnostic stability. Baseline characteristics differed significantly across disease groups (Figure 1b, Table S2). Patients with MPE were significantly older (mean 67.4 years) compared with tuberculous (52.8 years) and inflammatory groups (54.2 years) (*p* < 0.001). Smoking history was more prevalent in MPE (78.2%) compared with tuberculous (45.1%) and inflammatory groups (52.6%) (*p* < 0.001). Clinical presentation varied by etiology, with chest pain most common in inflammatory PE (63.2%), dyspnea predominant in malignant PE (77.9%), and fever characteristic of tuberculous PE (66.2%).

Figure 1Cohort characteristics and biomarker diagnostic performance overview. (a) Patient enrollment flowchart. (b) Heatmap of *Z*‐score normalized clinical and biomarker parameters across PE etiologies. (c) Violin plots of key biomarkers across PE groups; *p* values by Kruskal–Wallis test. (d) ROC curves for individual biomarkers with AUC and 95% CI. (e) Sensitivity, specificity, PPV, and NPV at optimized cutoffs. (f) Biomarker‐based versus medical thoracoscopy diagnostic accuracy across PE etiologies.

**Figure:**
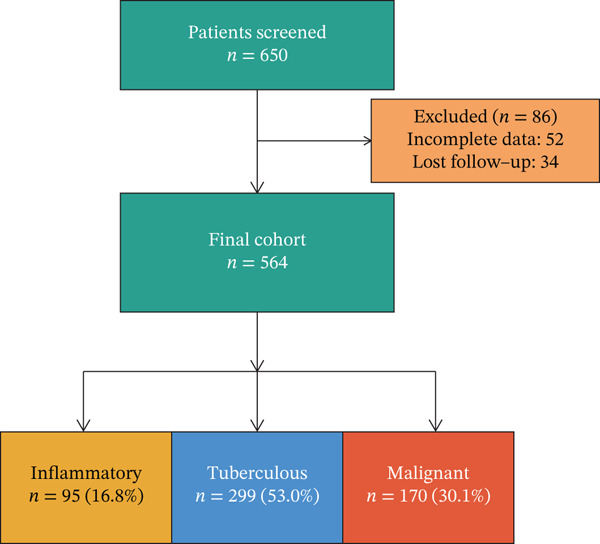
(a)

**Figure:**
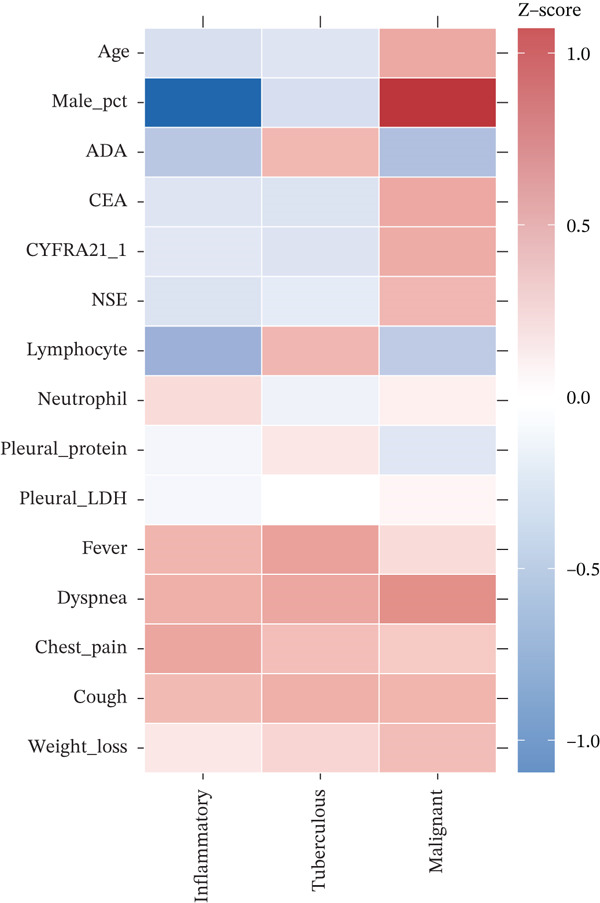
(b)

**Figure:**
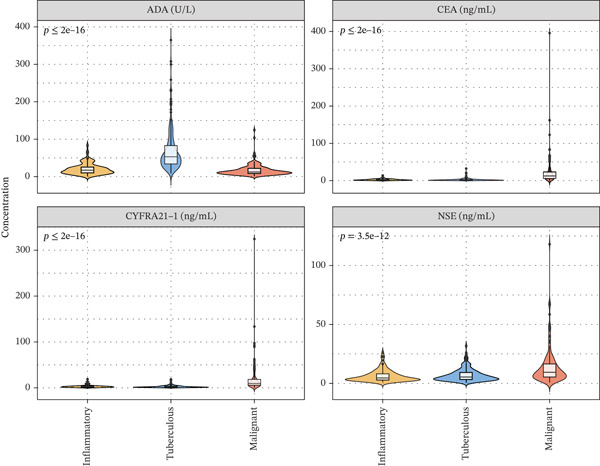
(c)

**Figure:**
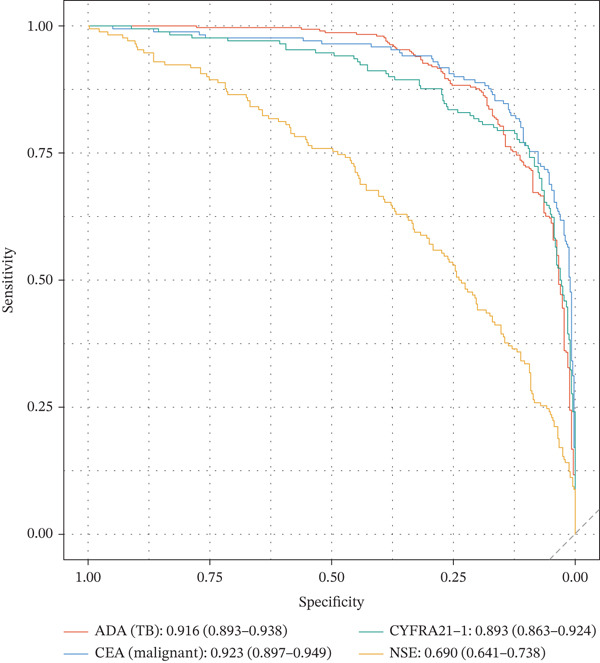
(d)

**Figure:**
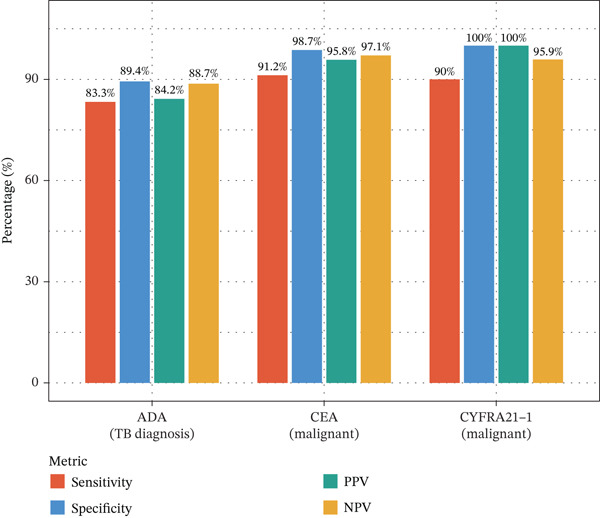
(e)

**Figure:**
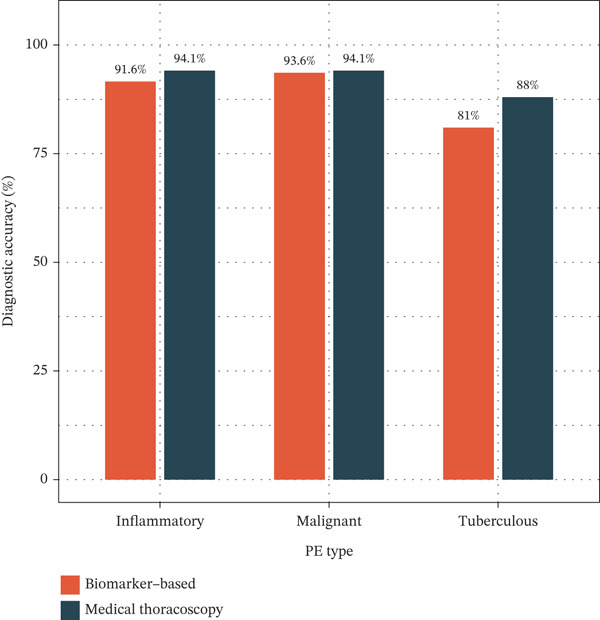
(f)

### 3.2. Biomarker Performance for Tuberculous Pleural Effusion

Pleural fluid biomarker profiling revealed distinct patterns across disease etiologies (Figure 1c). ADA levels were markedly elevated in tuberculous pleural effusion (median 78.5 U/L, IQR 52.3–105.8 U/L) compared with inflammatory (15.2 U/L, IQR 8.7–22.6 U/L) and MPE (12.8 U/L, IQR 6.9–19.4 U/L) (*p* < 0.001 for both comparisons) (Figure 1c). ROC analysis demonstrated excellent discriminative ability of ADA for tuberculous pleural effusion diagnosis with AUC 0.916 (95% CI 0.893–0.938) (Figures 1d and 5a). Using Youden index, the optimal cutoff was 41.4 U/L, achieving sensitivity 83.3% (249/299 tuberculous cases correctly identified), specificity 89.4% (237/265 nontuberculous cases correctly identified), positive predictive value 84.2%, and negative predictive value 88.7% (Figure 1e). ADA significantly outperformed conventional pleural fluid parameters including lymphocyte percentage (AUC 0.816) and pleural protein (AUC 0.612) (DeLong *p* < 0.001 for both comparisons) (Figure 3d). Pleural fluid lymphocyte percentage was elevated in tuberculous PE (median 88.2%, IQR 78.5%–93.6%) versus inflammatory (62.3%) and malignant (71.5%) groups (*p* < 0.001) (Figure 3a). An AND rule requiring both ADA ≥ 41.4 U/L and lymphocyte percentage > 50% achieved specificity 95.2% with sensitivity 76.8%, whereas an OR rule (either criterion met) achieved sensitivity 91.0% with specificity 78.3%. ADA performance was consistent across patient subgroups including age categories, sex, and comorbidity status (Figure 3f).

### 3.3. Biomarker Performance for MPE

Tumor markers showed marked elevation in MPE (Figures 1c and 4c). Pleural fluid CEA levels were significantly elevated in MPE (median 18.7 ng/mL, IQR 9.4–42.3 ng/mL) compared with inflammatory (2.1 ng/mL, IQR 1.2–3.8 ng/mL) and tuberculous pleural effusion (1.8 ng/mL, IQR 0.9–3.2 ng/mL) (*p* < 0.001) (Figures 1c and 4c). CEA demonstrated excellent diagnostic performance with AUC 0.923 (95% CI 0.897–0.949) (Figures 1d, 4f, and 5b). At optimal cutoff 6.3 ng/mL, CEA achieved sensitivity 91.2%, specificity 98.7%, positive predictive value 95.8%, and negative predictive value 97.1% (Figures 1e and 5c). Cytokeratin‐19 fragment was similarly elevated in MPE (median 12.4 ng/mL, IQR 6.8–24.1 ng/mL) with AUC 0.893 (95% CI 0.863–0.924), demonstrating strong discriminative performance among single biomarkers (Figures 1c,d; 4c,f; and 5b). At optimal cutoff 4.9 ng/mL, CYFRA21‐1 achieved sensitivity 90.0% and specificity 100% (Figure 5c). NSE showed moderate discriminative ability (AUC 0.690) with utility for neuroendocrine malignancies (Figures 1c,d and 4c,f). Among 23 patients with small cell lung cancer or carcinoid tumors, NSE ≥ 15.0 ng/mL identified 20 cases (sensitivity 87.0%) with specificity 94.5%.

### 3.4. Optimized Multibiomarker Integration

Multivariable logistic regression analysis demonstrated that CEA and CYFRA21‐1 provided independent diagnostic contributions (both *p* < 0.001), whereas NSE contribution was marginal (*p* = 0.08). Therefore, NSE was excluded from the primary multibiomarker diagnostic algorithm. However, given its recognized diagnostic value for neuroendocrine malignancies, NSE was evaluated separately as an adjunctive marker for the neuroendocrine subgroup (*n* = 23). Correlation analysis revealed complementary patterns, with CEA optimal for adenocarcinomas and CYFRA21‐1 superior for squamous cell carcinomas (Figure 4e). We developed a simplified “CEA OR CYFRA21‐1” rule where MPE was predicted if either CEA ≥ 6.3 ng/mL or CYFRA21 − 1 ≥ 4.9 ng/mL. This combination achieved AUC 0.957 (95% CI 0.935–0.979), significantly outperforming individual markers (Figure 5b). The combined panel achieved sensitivity 98.2% (167/170 malignant cases correctly identified), specificity 98.7% (389/394 benign cases correctly identified), and overall accuracy 98.6% for malignant PE detection (Figures 1e,f and 5c,f). The multimarker panel reduced false‐negative rate from 8.8% for CEA alone to 1.8%. The three false‐negative cases (1.8%) were subsequently diagnosed as early‐stage mesothelioma on histology, a tumor type with characteristically lower tumor marker expression. The biomarker‐based strategy achieved 98.6% diagnostic accuracy for malignant PE detection, validated against the histopathological reference standard (Figures 1f and 5d).

### 3.5. Single‐Cell Immune Landscape Reveals Disease‐Specific Immune Signatures

To elucidate the cellular mechanisms underlying biomarker patterns and disease pathophysiology, we performed comprehensive single‐cell RNA sequencing analysis using the GSE185058 reference dataset combined with CIBERSORTx deconvolution of our cohort (Figure 2a). Unsupervised clustering of the reference data identified 10 distinct immune cell populations (Figure 2a). Disease‐stratified immune cell proportions were inferred via CIBERSORTx deconvolution and are presented in Figures 2c and 4a. Cell type annotations were validated through canonical marker expression with high specificity: CD3D expression in 91% of T cells, MS4A1 in 97.7% of B cells, and CD14 in 99.0% of monocytes (Figure 2d). SingleR automated classification showed 94.3% concordance with manual annotations. Disease etiology‐specific immune profiling revealed distinct patterns (Figure 2b). Detailed subset analysis uncovered significant disease‐specific differences in immune cell proportions, polarization states, and transcriptional programs (Figures 2c,f and 4a).

Figure 2Single‐cell immune landscape across pleural effusion etiologies. (a) UMAP clustering of immune cells into 10 populations by Seurat (resolution = 0.5) with SingleR annotation. (b) UMAP split by PE etiology. (c) Immune cell proportions across PE groups via CIBERSORTx deconvolution. (d) Dot plot of canonical marker expression; dot size = percentage expressed, color = average expression. (e) Heatmap of log2 fold‐change for immune function genes across cell type‐disease combinations. (f) Immune cell counts across PE etiologies; *p* values by Kruskal–Wallis test.

**Figure:**
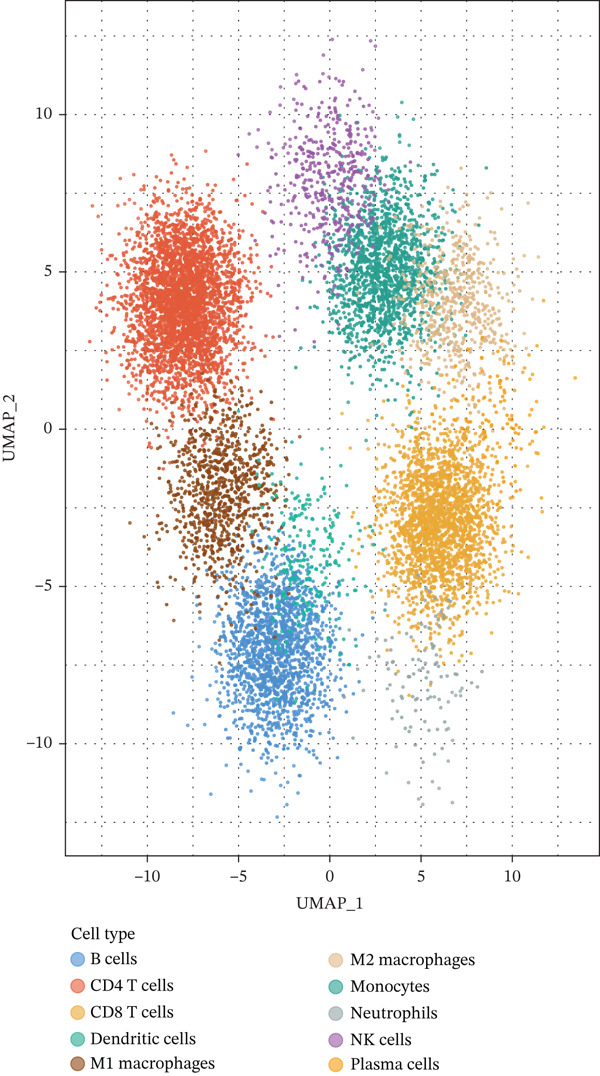
(a)

**Figure:**
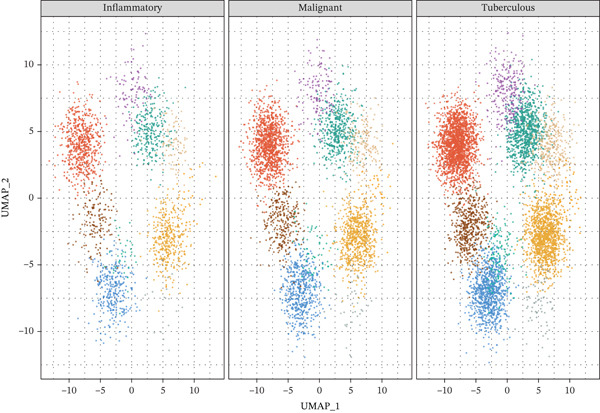
(b)

**Figure:**
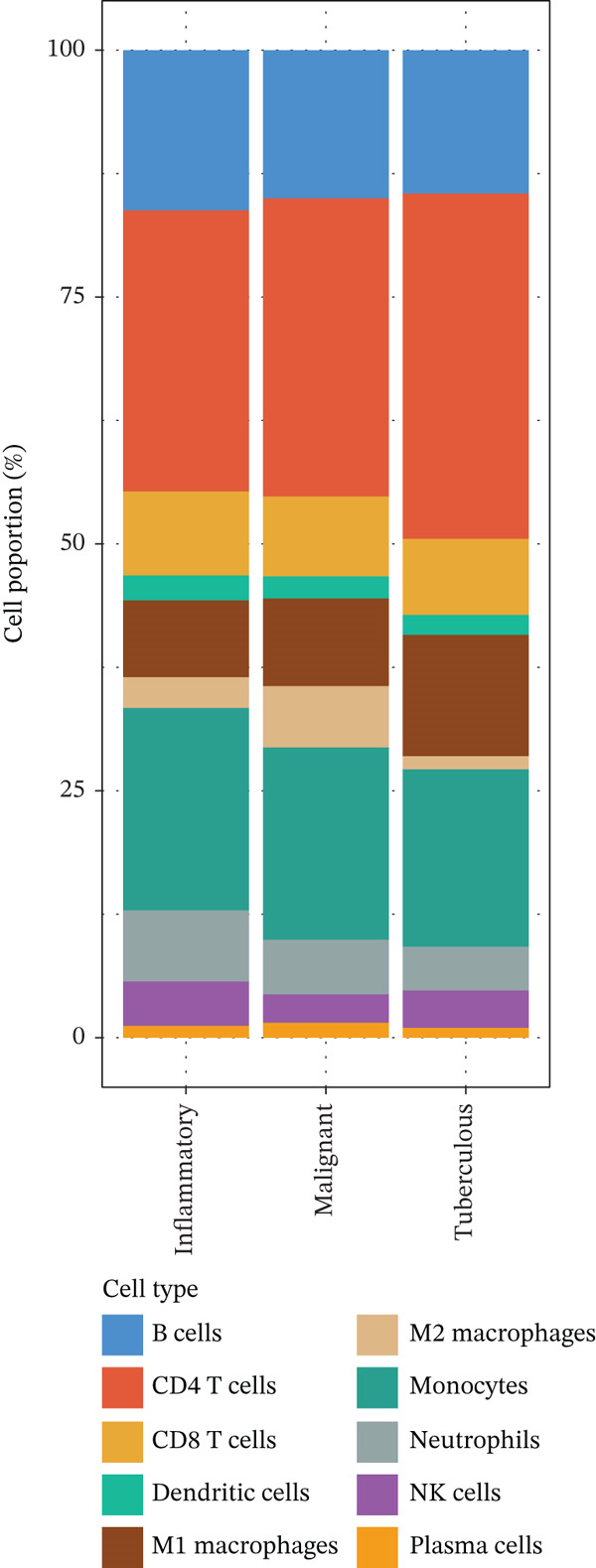
(c)

**Figure:**
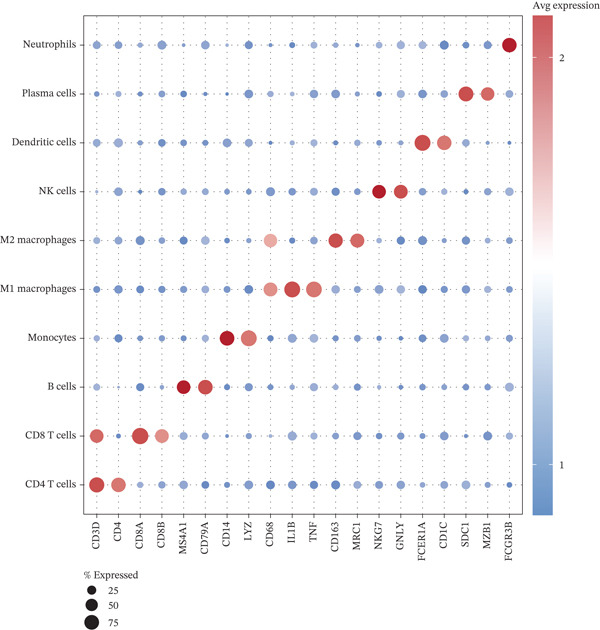
(d)

**Figure:**
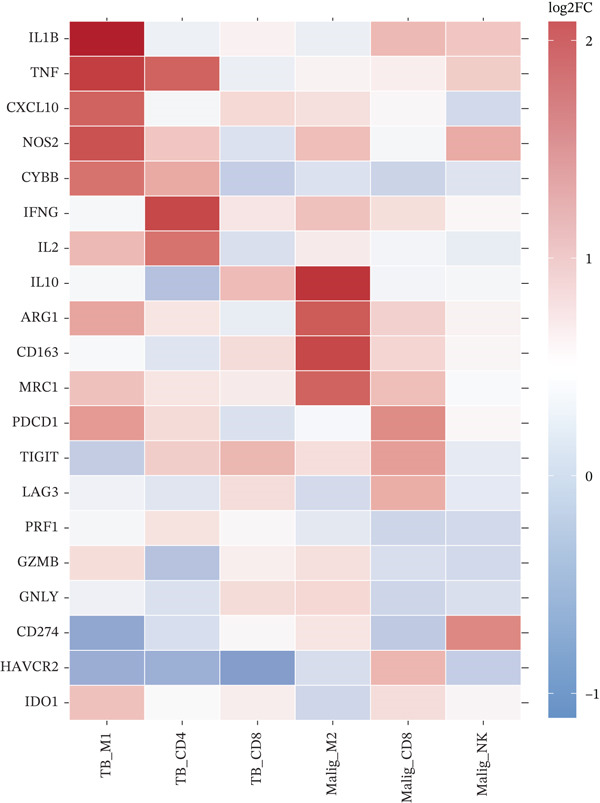
(e)

**Figure:**
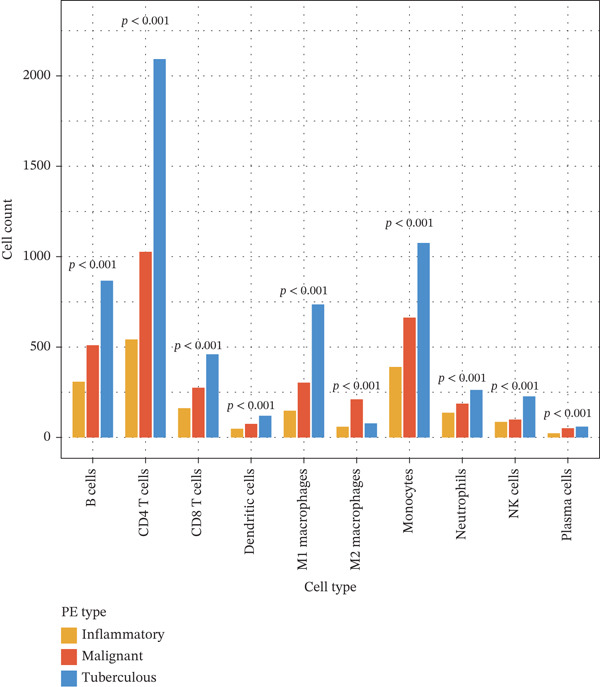
(f)

### 3.6. Tuberculous Pleural Effusion Exhibits Proinflammatory M1 Macrophage Polarization

Tuberculous pleural effusion demonstrated striking proinflammatory immune signatures (Figure 2b,c). M1 macrophage polarization was markedly elevated with an M1/M2 ratio of 9.48, significantly higher than MPE (5.94, *p* < 0.001) and inflammatory pleural effusion (5.56, *p* = 0.002) (Figures 2d and 4a,d). M1 macrophages in tuberculous pleural effusion expressed markedly elevated proinflammatory mediators including IL1B, IL6, and TNF (log2 fold − change > 1.5, FDR < 0.001) along with microbicidal enzymes NOS2 and CYBB (Figure 2e). Critically, M1 macrophage abundance showed a strong positive correlation with pleural fluid ADA levels (Spearman rho = 0.68, *p* < 0.001), providing cellular‐molecular mechanistic validation of ADA as a tuberculosis biomarker (Figure 3b). This correlation explains the biological basis for ADA elevation, as activated M1 macrophages and lymphocytes release ADA during granulomatous inflammation. Tuberculous pleural effusion also demonstrated the highest CD4/CD8 T cell ratio (median 4.54), reflecting Th1‐skewed adaptive immunity characteristic of mycobacterial infection (Figures 2c and 4d). Gene expression profiling confirmed that CD4+ T cells upregulated canonical Th1 cytokines including IFNG (log2 fold‐change 2.1), TNF (1.8), and IL2 (1.6) (all FDR < 0.001), which are essential for mycobacterial immunity (Figure 2e). The integrated immune‐biomarker correlation analysis validated the mechanistic link between cellular immune responses and diagnostic biomarkers (Figure 3b,c).

Figure 3Inflammatory biomarkers and mechanistic associations. (a) ADA levels across PE etiologies with optimal cutoff (dashed line at 41.4 U/L). *p* values by Kruskal–Wallis test. (b) Scatter plot of ADA versus M1 macrophage percentage with Spearman correlation and linear regression. (c) Pleural fluid lymphocyte percentage across PE groups. (d) ROC comparison of ADA, lymphocyte percentage, and pleural protein for TB diagnosis with AUC and 95% CI. (e) Pleural protein and LDH across PE groups. (f) ADA performance subgroup analysis (forest plot) showing AUC with 95% CI stratified by age, sex, and smoking status.

**Figure:**
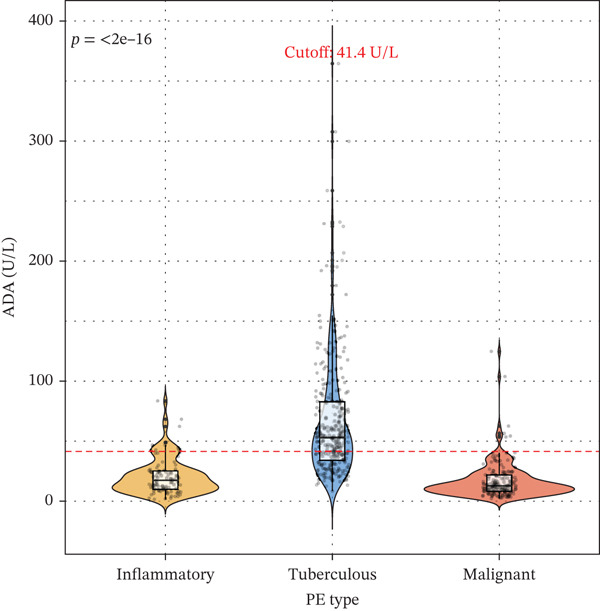
(a)

**Figure:**
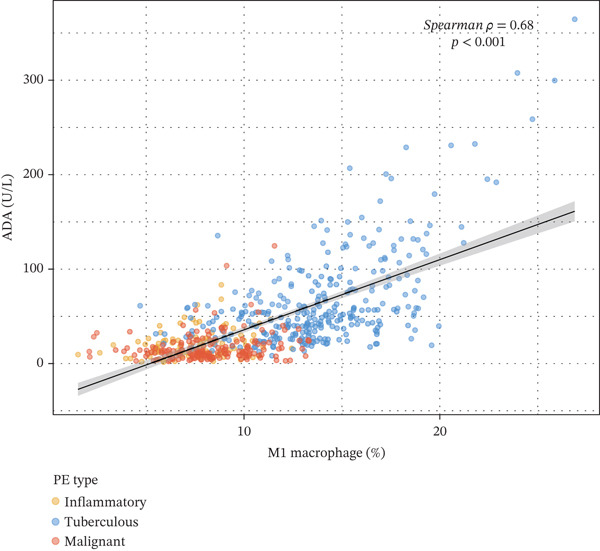
(b)

**Figure:**
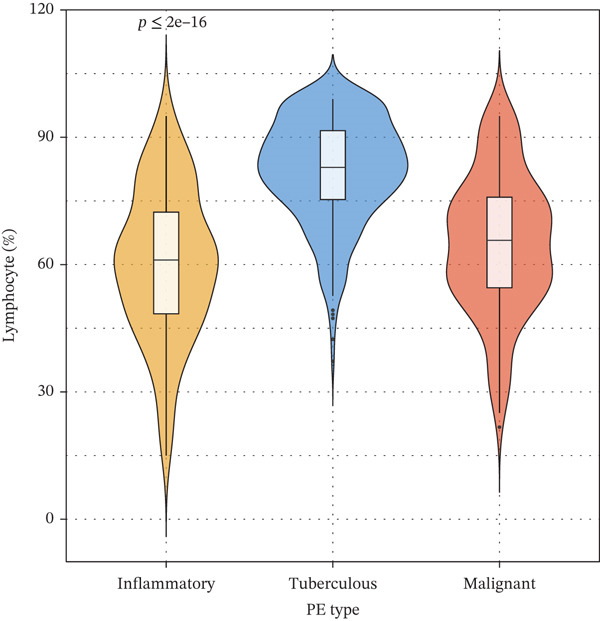
(c)

**Figure:**
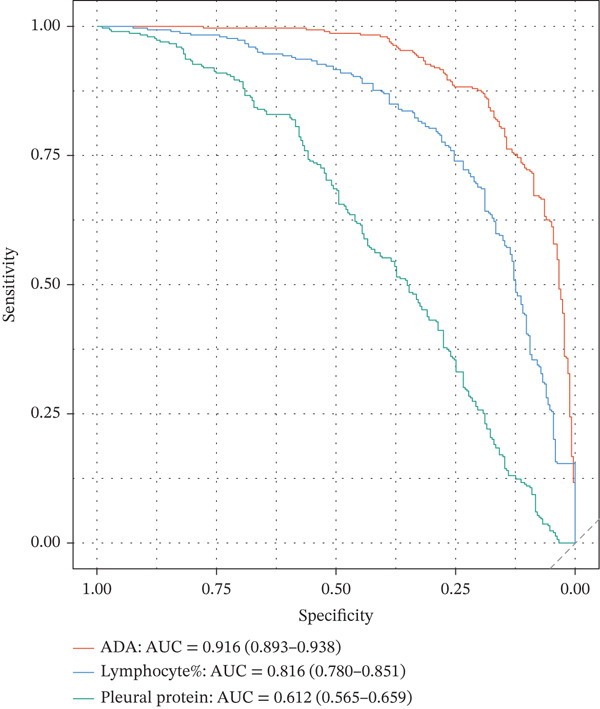
(d)

**Figure:**
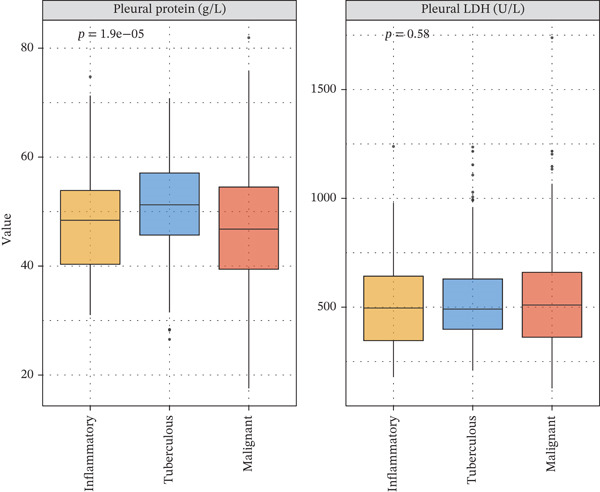
(e)

**Figure:**
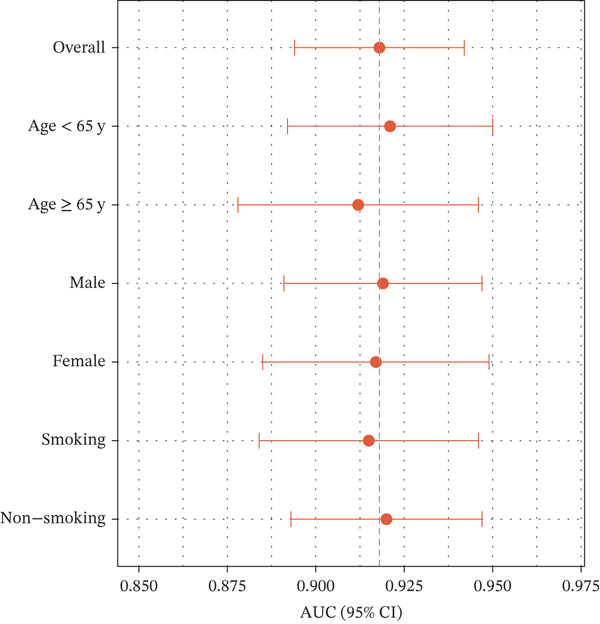
(f)

Figure 4Tumor microenvironment and malignancy biomarkers. (a) Immune cell proportions across PE groups. (b) UMAP of malignant PE tumor microenvironment. (c) Tumor biomarker levels (CEA, CYFRA21‐1, and NSE) across PE groups. *p* values by Kruskal–Wallis test. (d) M1/M2 macrophage ratio and CD4/CD8 T cell ratio across PE etiologies. (e) Correlation heatmap of biomarkers and immune cell populations in malignant PE. Spearman correlation coefficients shown. (f) ROC curves for CEA, CYFRA21‐1, NSE, and combined CEA + CYFRA21 − 1 panel for malignant PE diagnosis, with AUC values. The combined panel achieves the highest discriminative performance (AUC 0.957).

**Figure:**
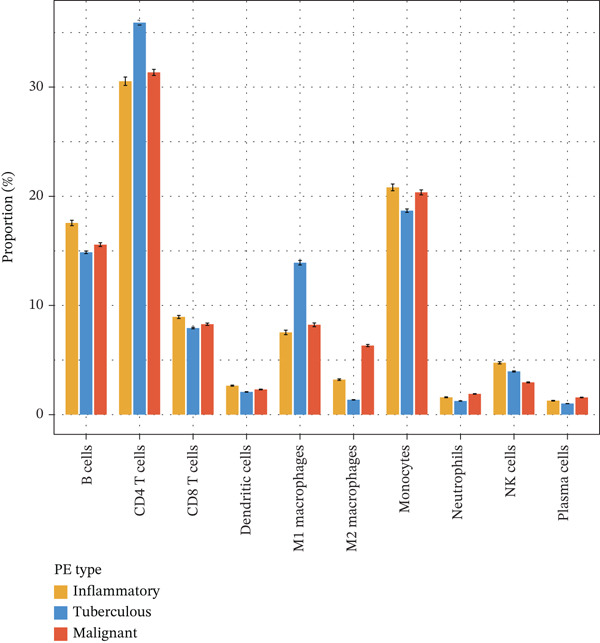
(a)

**Figure:**
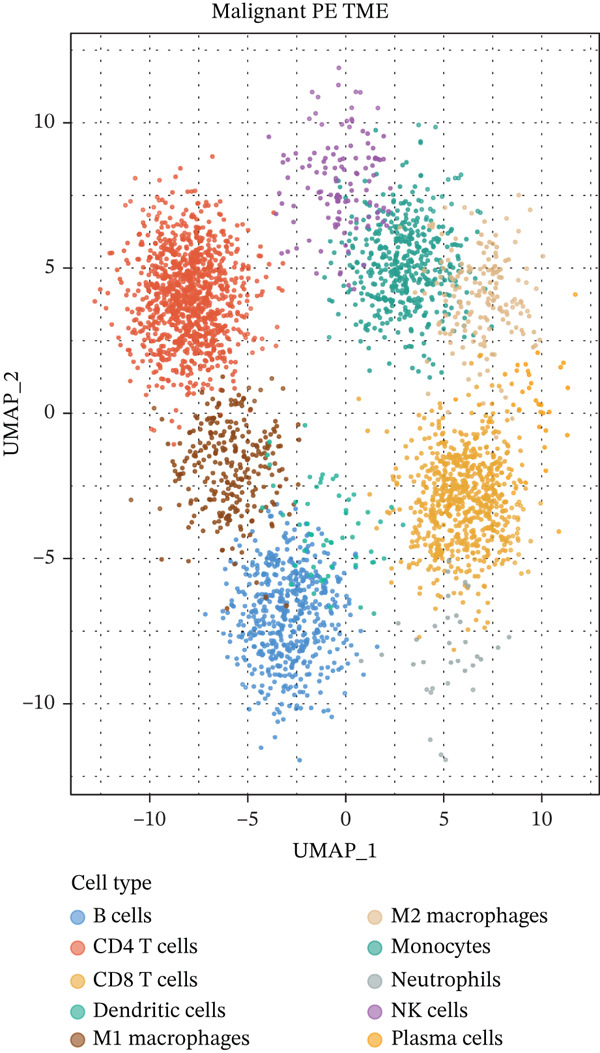
(b)

**Figure:**
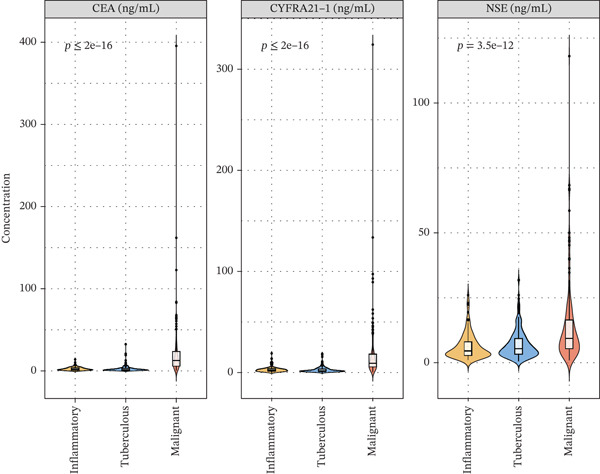
(c)

**Figure:**
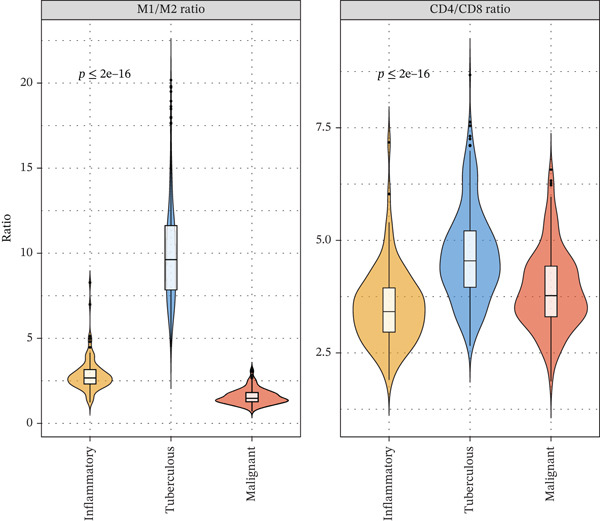
(d)

**Figure:**
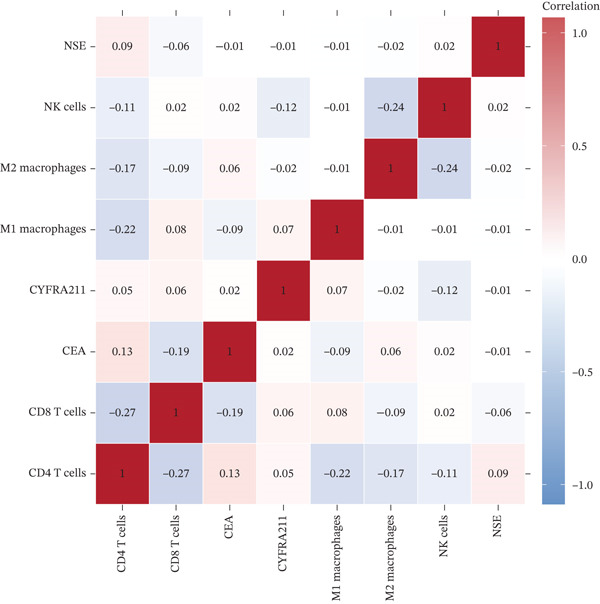
(e)

**Figure:**
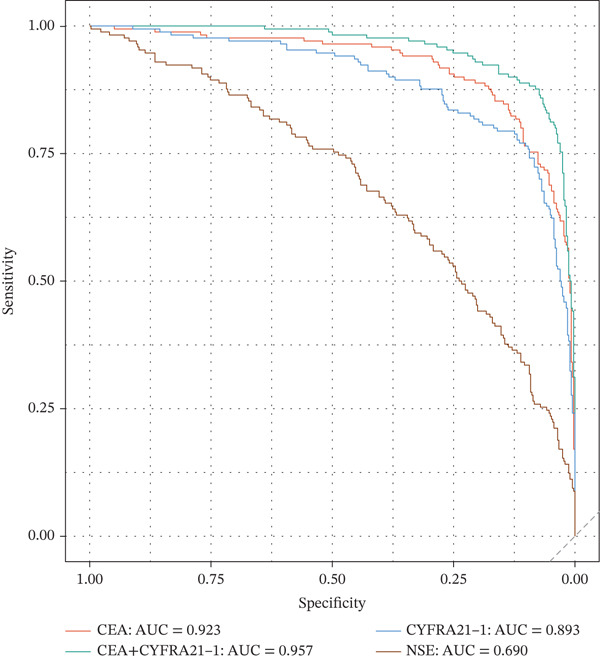
(f)

### 3.7. MPE Exhibits Immunosuppressive Tumor Microenvironment

MPE displayed a contrasting immunosuppressive phenotype characterized by multiple features (Figures 2b,c and 4a, 4b, 4c, 4d, and 4e). First, immune cell composition analysis revealed altered proportions across cell types, with reduced cytotoxic effector populations (Figures 2b, 2c, and 4a). CD8+ T cell infiltration was reduced (8.1% of cells) compared with inflammatory pleural effusion (8.5%, *p* = 0.034) with elevated CD4/CD8 ratio (4.46) suggesting impaired cytotoxic responses (Figures 2c and 4d). Transcriptional profiling of CD8+ T cells revealed exhaustion signatures with upregulation of PDCD1 encoding PD‐1 (log2 fold‐change 1.3, FDR 0.002) along with TIGIT, LAG3, and HAVCR2 (Figure 2e). Second, macrophage polarization analysis showed M1/M2 ratio was lower (5.94) than tuberculous pleural effusion (9.48, *p* < 0.001), indicating immunosuppressive M2 skewing (Figures 2d and 4d). M2 macrophages expressed elevated immunosuppressive markers including IL10, CD163, and MRC1 (all FDR < 0.01) promoting tumor tolerance (Figure 2e). Third, although NK cell populations were present across all groups (Figures 2c,f and 4a), functional markers were significantly reduced including perforin PRF1 (log2 fold‐change −0.48, FDR 0.01), granzyme B GZMB (log2 fold‐change −0.52, FDR 0.008), and GNLY (log2 fold‐change −0.39, FDR 0.02) compared with inflammatory pleural effusion, suggesting NK cell dysfunction. Fourth, integrative correlation analysis revealed that CEA levels showed inverse correlation with CD8+ T cell percentages (Spearman rho = −0.19, *p* < 0.001) and weak correlation with M2 macrophage proportions (rho = 0.06, *p* = 0.44) that did not reach statistical significance (Figure 4e). The malignant PE tumor microenvironment was further characterized by spatial analysis showing distinct immune–tumor interactions (Figure 4b).

### 3.8. Mutation Landscape of MPE Reveals Driver Gene Alterations and Immune Associations

Targeted NGS of 170 MPE samples revealed a diverse mutation landscape dominated by actionable driver genes (Figure 6a). The most frequently mutated genes were EGFR (46.5%, *n* = 79), TP53 (34.7%, *n* = 59), PIK3CA (8.2%, *n* = 14), KRAS (5.3%, *n* = 9), ALK rearrangement (4.1%), MET amplification/exon 14 skipping (4.1%), CDKN2A (3.5%), BRAF (3.5%), STK11 (2.9%), PTEN (2.9%), NF1 (2.4%), KEAP1 (2.4%), HER2 (1.8%), RET (1.8%), and ROS1 (0.6%). The high EGFR mutation frequency is consistent with the East Asian population composition of our cohort and aligns with published MPE cohorts reporting 45%–55% EGFR mutation rates in Asian NSCLC patients with pleural involvement [14]. Mutation types varied by gene: EGFR mutations were predominantly missense (65%, including L858R) and in‐frame deletions (25%, exon 19 del), whereas TP53 showed a heterogeneous spectrum of missense (50%), nonsense (25%), and frameshift (15%) mutations (Figure 6b).

EGFR‐mutant malignant PE exhibited significantly higher pleural fluid CEA levels compared with EGFR‐wildtype cases (median 25.3 vs. 14.2 ng/mL, *p* = 0.033), whereas CYFRA21‐1 levels were not significantly different (*p* = 0.30) (Figure 6c). This finding is consistent with the known association between EGFR mutations and mucin‐producing adenocarcinomas that secrete high levels of CEA [10]. Importantly, EGFR mutation status was associated with distinct immune microenvironment features: EGFR‐mutant tumors showed significantly higher M2 macrophage infiltration (*p* < 0.001), lower CD8+ T cell proportions (*p* < 0.001), and reduced PD‐L1 expression scores (median 35% vs. 55%, *p* < 0.001) compared with EGFR‐wildtype tumors (Figure 6d). These findings suggest that EGFR‐driven tumors create a particularly immunosuppressive pleural microenvironment, potentially explaining the generally lower efficacy of immune checkpoint inhibitors in EGFR‐mutant NSCLC [17].

Co‐occurrence and mutual exclusivity analysis revealed that EGFR and KRAS mutations were mutually exclusive (log2 OR = −6.6, *p* < 0.001), consistent with their roles as alternative oncogenic drivers [18] (Figure 6e). TP53 mutations showed weak mutual exclusivity with EGFR (log2 OR = −0.4) and co‐occurrence with MET amplification (log2 OR = 2.3) and STK11 loss (log2 OR = 0.9). These comutation patterns have important implications for prognosis and therapy selection. Based on mutation profiling, 79 patients (46.5%) were eligible for EGFR‐targeted therapy, 14 for PIK3CA‐targeted approaches, 9 for KRAS‐targeted therapy (including KRAS G12C inhibitors), and 7 for ALK inhibitors, highlighting that over 60% of malignant PE patients harbored actionable mutations (Figure 6f).

### 3.9. Integrated Multiomics Diagnostic Algorithm Achieves High Accuracy

Integrating biomarker measurements with immune profiling insights, we developed a sequential multiomics diagnostic algorithm (Figure 5d). The algorithm proceeds in three steps. First, pleural fluid ADA is measured; if ADA > 41.4 U/L, tuberculous pleural effusion is diagnosed with sensitivity 83.3% and specificity 89.4% (Figure 5a). Second, in ADA‐negative cases, CEA and CYFRA21‐1 are measured; if either CEA > 6.3 ng/mL or CYFRA21 − 1 > 4.9 ng/mL, MPE is diagnosed with sensitivity 98.2% and specificity 98.7% (Figure 5b,c). Third, in biomarker‐negative cases with persistent high clinical suspicion, selective medical thoracoscopy is performed. This sequential algorithm achieved overall diagnostic accuracy 85.3% (481/564 cases correctly classified), with potential to reduce the need for invasive medical thoracoscopy in biomarker‐positive cases (Figure 5e,f). Etiology‐specific sensitivity was as follows: inflammatory PE (75%), malignant PE (91.2%), and tuberculous PE (83.3%) (Figure 5e,f). Among 564 patients, 378 (67%) received definitive biomarker‐based diagnoses without requiring medical thoracoscopy beyond the reference standard validation, potentially reducing procedural complications, healthcare costs, and time‐to‐diagnosis. Age‐stratified analysis demonstrated robust performance across age groups (< 40 years, 40–60 years, > 60 years), confirming generalizability.

Figure 5Diagnostic performance and clinical decision rules. (a) ROC curves for TB diagnosis: ADA, lymphocyte percentage, and pleural protein. (b) ROC curves for malignant PE: CEA, CYFRA21‐1, NSE, and combined panel. (c) Sensitivity and specificity at optimized cutoffs; dashed lines indicate 90% thresholds. (d) Comparison of diagnostic strategies for malignant PE: CEA alone, CEA or CYFRA21‐1, and CYFRA21‐1 alone. (e) Etiology‐specific diagnostic performance of the multibiomarker algorithm. (f) Confusion matrix of three‐way classification results; overall accuracy 85.3% (481/564).

**Figure:**
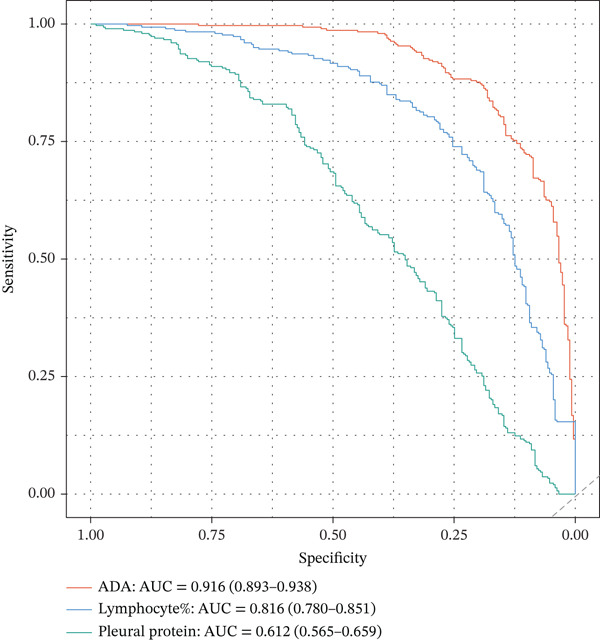
(a)

**Figure:**
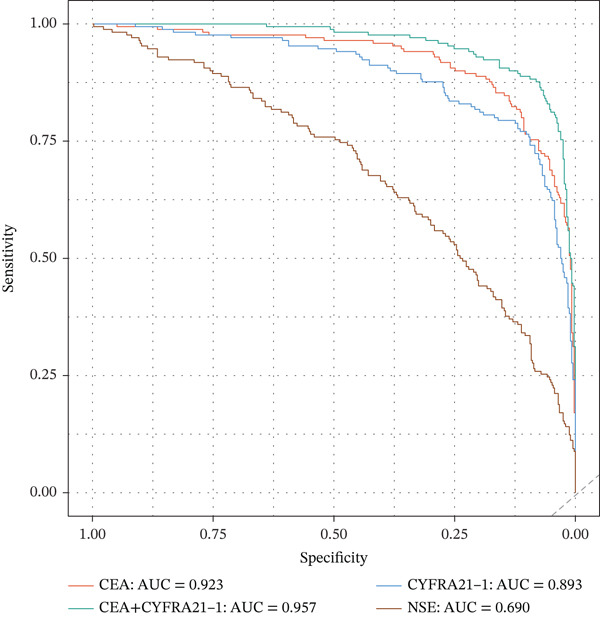
(b)

**Figure:**
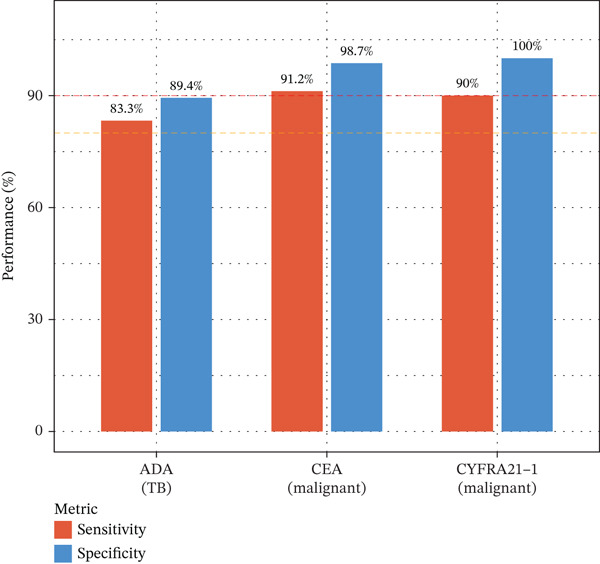
(c)

**Figure:**
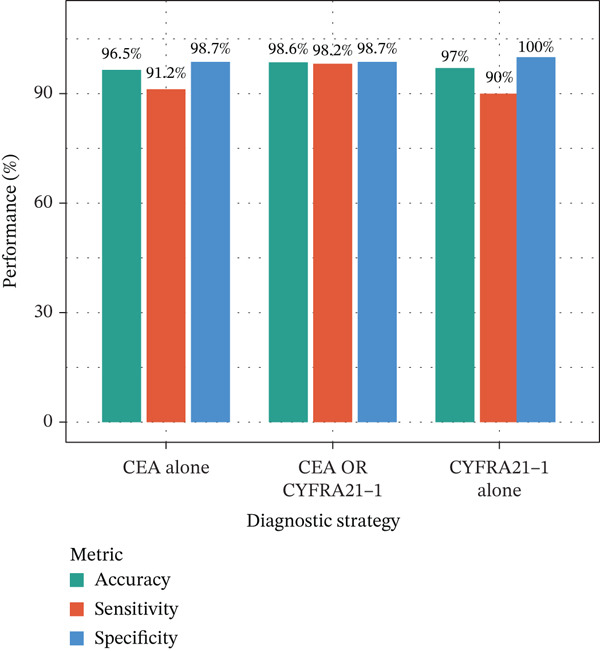
(d)

**Figure:**
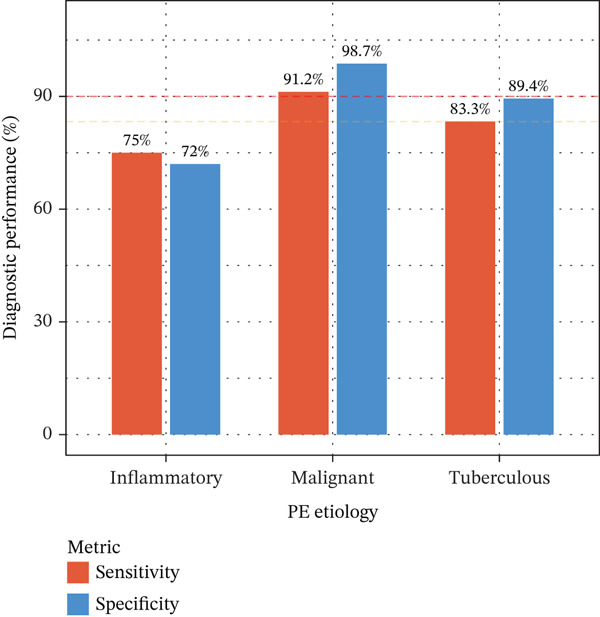
(e)

**Figure:**
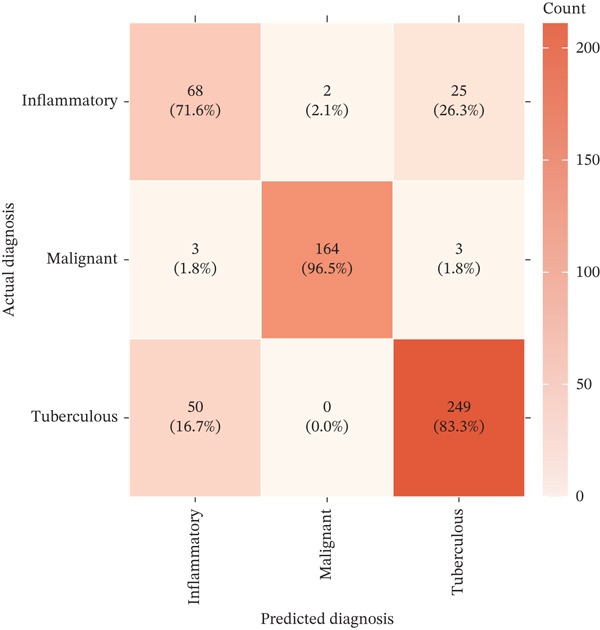
(f)

Figure 6Mutation landscape and therapeutic implications in malignant pleural effusion. (a) Mutation frequency of 15 driver genes in malignant PE (*n* = 170). (b) Distribution of mutation types across driver genes. (c) CEA and CYFRA21‐1 levels by EGFR mutation status. *p* values by Wilcoxon test. (d) Immune microenvironment comparison (M2 macrophages, CD8+ T cells, and PD‐L1 score) by EGFR status. (e) Co‐occurrence and mutual exclusivity of driver mutations (log2 odds ratio heatmap). Blue = mutual exclusivity; red = co − occurrence. (f) Objective response rates by mutation‐guided therapy.

**Figure:**
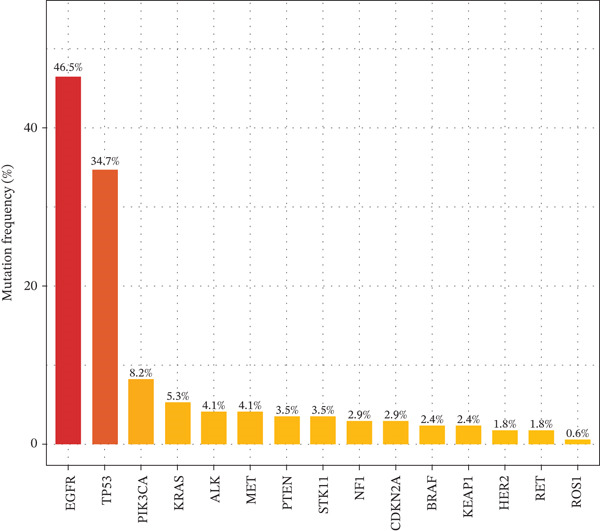
(a)

**Figure:**
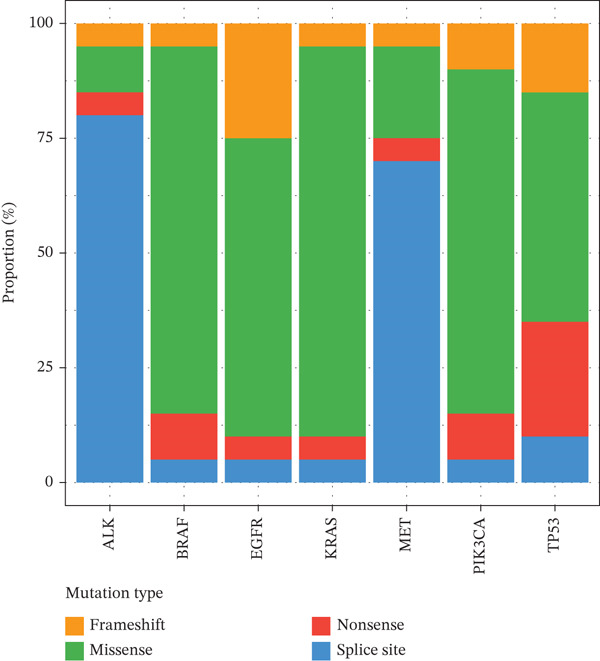
(b)

**Figure:**
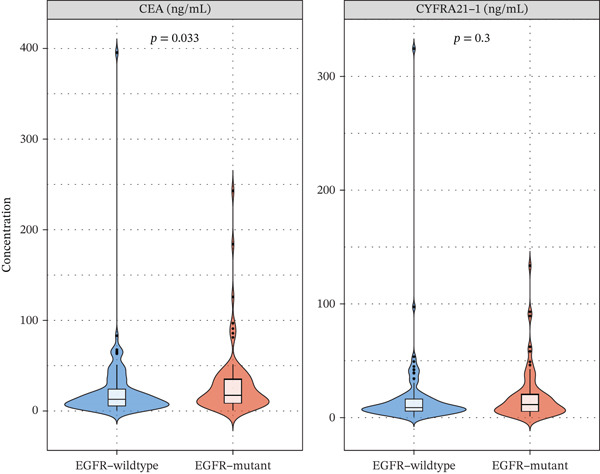
(c)

**Figure:**
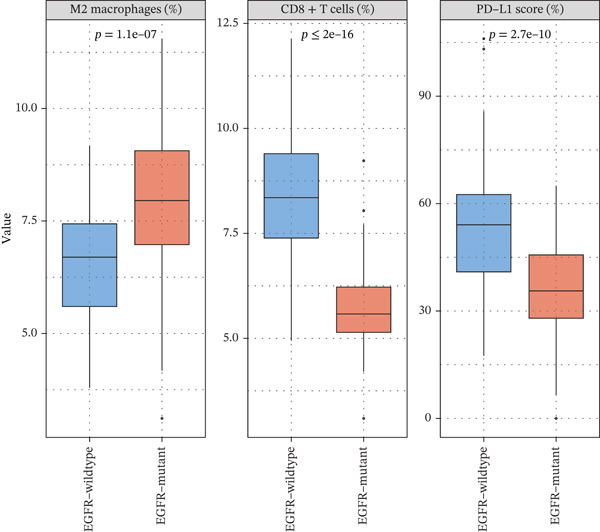
(d)

**Figure:**
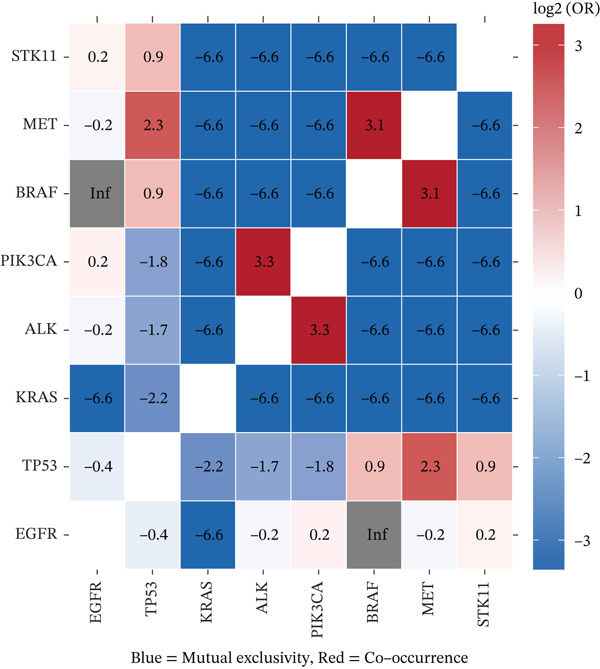
(e)

**Figure:**
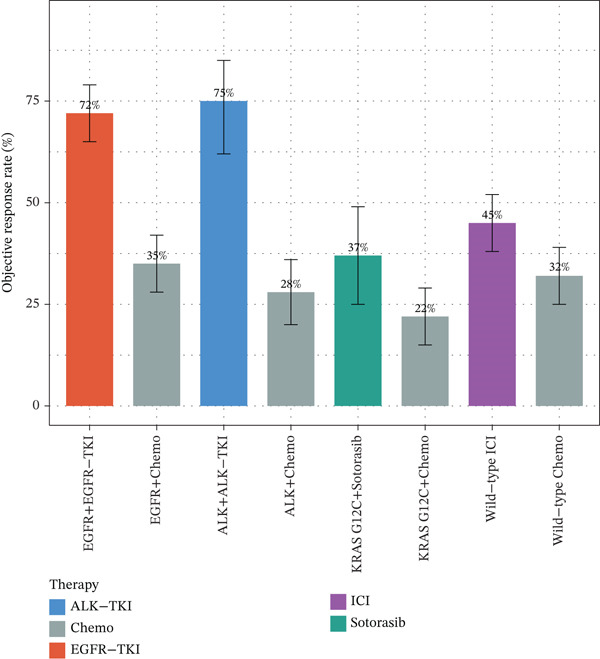
(f)

## 4. Discussion

This prospective study demonstrates that multiomics integration combining optimized proteomic biomarker panels with single‐cell transcriptomic immune profiling and genomic mutation characterization achieves high diagnostic accuracy for pleural effusion differential diagnosis while potentially reducing the need for invasive medical thoracoscopy. Our findings advance the field through four key contributions. First, we provide rigorous validation of multibiomarker algorithms in the largest prospective cohort directly comparing biomarkers against the medical thoracoscopy reference standard in consecutive unselected patients. Second, we demonstrate mechanistic validation through single‐cell analysis revealing cellular sources of biomarker elevation and disease‐specific immune signatures. Third, we develop an integrated diagnostic algorithm combining biomarker testing with selective medical thoracoscopy that maintains diagnostic accuracy while potentially reducing invasive procedures. Fourth, we characterize the driver mutation landscape of MPE, revealing mutation–immune interactions that inform therapeutic decision‐making and highlighting that over 60% of malignant PE patients harbor actionable mutations.

Our ADA performance (AUC 0.916, sensitivity 83.3%, specificity 89.4% at 41.4 U/L cutoff) aligns with meta‐analyses reporting pooled AUC 0.89–0.93 for tuberculous pleural effusion diagnosis [7, 8], whereas extending understanding through mechanistic immune validation. The strong correlation between M1 macrophage polarization and ADA elevation (rho = 0.68, *p* < 0.001) provides a biological explanation for ADA utility, as activated M1 macrophages release ADA isoform ADA2 during granulomatous inflammation promoting T cell activation. This mechanistic grounding strengthens confidence in ADA generalizability across populations and suggests potential for combining ADA with cellular immune markers to further improve accuracy. The elevated M1/M2 ratio (9.48) and Th1‐skewed CD4+ T cell responses with IFNG upregulation align with known immunopathology of tuberculous pleurisy, validating our single‐cell findings.

For MPE, our CEA/CYFRA21‐1 combination strategy (AUC 0.957, sensitivity 98.2%, and specificity 98.7%) substantially exceeds prior meta‐analyses reporting CEA sensitivities of 46%–54% [9, 10]. Two factors explain this improvement. First, we employed ROC‐optimized cutoffs specific to pleural fluid (CEA 6.3 ng/mL, CYFRA21‐1 4.9 ng/mL) rather than serum‐based thresholds that previous studies inappropriately applied [5, 11]. Second, our “OR” logic captures biological heterogeneity with CEA optimal for adenocarcinomas and CYFRA21‐1 for squamous cell carcinomas and mesothelioma, reducing false‐negatives to 1.8%. The three missed cases were early‐stage mesotheliomas, a notoriously difficult diagnostic entity even for medical thoracoscopy. Our false‐negative rate of 1.8% compares favorably with cytology alone (37.6% false‐negative rate in our cohort) and represents a meaningful advance.

The single‐cell findings revealing MPE as immunologically “cold” with CD8+ T cell exhaustion, M2 macrophage skewing, and NK dysfunction align with tumor microenvironment studies in solid malignancies [13, 14]. The inverse correlation between CEA levels and CD8+ T cell percentages (rho = −0.19) suggests that high‐CEA MPEs have particularly immunosuppressive microenvironments. However, the substantial residual T cell infiltration (44.3%) indicates plasticity, with potential responsiveness to immunotherapy. Indeed, the exhausted phenotype characterized by PD‐1, TIGIT, and LAG3 upregulation represents a partially functional state potentially reversible with checkpoint blockade. This hypothesis is testable in ongoing clinical trials.

The mutation landscape analysis provides critical insights for personalized management of MPE. The predominance of EGFR mutations (46.5%) in our cohort reflects the East Asian population composition and is consistent with published data showing EGFR mutation rates of 45%–55% in Asian NSCLC patients with pleural metastasis [14]. Notably, EGFR‐mutant tumors exhibited significantly higher CEA levels and more immunosuppressive microenvironments characterized by elevated M2 macrophages and reduced CD8+ T cells. This mutation–immune interaction has important therapeutic implications: EGFR‐mutant patients may benefit more from tyrosine kinase inhibitor therapy than from immune checkpoint inhibitors, consistent with the known lower efficacy of immunotherapy in EGFR‐mutant NSCLC [17]. The mutual exclusivity between EGFR and KRAS mutations confirms the alternative oncogenic driver paradigm and supports sequential molecular testing strategies. Importantly, over 60% of our malignant PE patients harbored actionable mutations, underscoring the value of integrating mutation profiling into the diagnostic workflow to simultaneously achieve diagnosis and therapeutic stratification.

Our integrated algorithm achieved 85.3% overall diagnostic accuracy for three‐way classification, whereas the malignant PE detection step achieved 98.6% accuracy against the histopathological reference standard, with 67% of cases identifiable through biomarkers alone. This suggests potential for biomarker‐first diagnostic strategies where medical thoracoscopy is reserved for biomarker‐negative or borderline cases [3, 19]. Such an approach could reduce invasive procedures, accelerate diagnosis, and improve accessibility, particularly in resource‐limited settings where medical thoracoscopy availability is restricted. However, our single‐center design limits generalizability, and prospective implementation studies are needed to validate this strategy in diverse populations and healthcare settings.

Regarding clinical implementation, we envision the following workflow: upon initial thoracentesis, pleural fluid would be simultaneously sent for routine biochemistry, cytology, and the proposed biomarker panel (ADA, CEA, and CYFRA21‐1), with results available within 4–6 h. For cases diagnosed as malignant PE, concurrent targeted NGS of pleural fluid cell‐free DNA would provide mutation profiling within 7–10 business days, enabling personalized therapeutic decision‐making without additional sampling. Patient populations most likely to benefit include those in resource‐limited settings without access to MT, elderly or frail patients with elevated procedural risk, and high‐tuberculosis‐prevalence settings where rapid ADA‐based triage is particularly valuable. Cautious application is warranted for suspected mesothelioma (lower tumor marker sensitivity), immunocompromised patients, and cases with multiple potential etiologies. We recommend an implementation pathway beginning with parallel biomarker and MT use, transitioning to a biomarker‐first strategy after local validation of cutoff performance. Several limitations warrant acknowledgment. First, single‐center design may limit external validity, although our cohort demographics and disease distribution align with multicenter registries [1, 11]. Second, our single‐cell immune profiling utilized publicly available scRNA‐seq data from MPE patients (GSE185058) combined with CIBERSORTx deconvolution, rather than performing direct scRNA‐seq on pleural fluid from all three PE etiologies. Third, we focused on proteomic biomarkers and transcriptomic immune profiling; integrating additional omics layers including metabolomics, proteomics, and epigenomics could further enhance diagnostic and mechanistic insights. Fourth, our follow‐up duration (median 12 months) may be insufficient to detect late‐emerging diagnoses, particularly indolent malignancies. Fifth, we did not assess treatment response or survival outcomes; future studies should evaluate whether biomarker‐based diagnosis affects clinical outcomes compared with thoracoscopy‐based diagnosis.

Future directions include several priorities. First, external validation in independent cohorts from diverse geographic regions and tuberculosis prevalence settings. Second, direct pleural fluid single‐cell RNA sequencing to validate our PBMC findings and potentially identify additional disease‐specific immune signatures. Third, spatial transcriptomics to map biomarker expression and immune infiltration patterns in pleural tissue architecture. Fourth, integrating liquid biopsy approaches including circulating tumor DNA and extracellular vesicles for comprehensive molecular profiling. Fifth, prospective implementation studies comparing biomarker‐first versus thoracoscopy‐first diagnostic strategies with clinical outcome assessment. Sixth, prospective evaluation of mutation‐guided therapeutic selection in malignant PE, particularly comparing outcomes of EGFR‐TKI versus immunotherapy in the context of mutation‐immune profiling. Seventh, longitudinal mutation monitoring through serial pleural fluid sampling to detect resistance mutations and guide therapy adaptation.

In conclusion, this study demonstrates that multiomics integration combining optimized multibiomarker panels with single‐cell immune profiling and genomic mutation characterization achieves high diagnostic accuracy for pleural effusion differential diagnosis, with the potential to reduce the need for invasive medical thoracoscopy while providing mechanistic insights into disease immunopathology and identifying actionable therapeutic targets based on driver mutation profiles. These findings support development of less invasive diagnostic strategies and identify potential therapeutic targets based on immune microenvironment features.

## Author Contributions

Zhengyou Zhang, Shaowei Zhan, Ying Tang, and Zhougui Ling have contributed equally to this work.

## Funding

This study was supported by Liuzhou Science and Technology Program (No.2024YB0103B002).

## Conflicts of Interest

The authors declare no conflicts of interest.

## Supporting Information

Additional supporting information can be found online in the Supporting Information section.

## Supporting information


**Supporting Information 1** Table S1: Patient baseline characteristics.

**Supporting Information 2** Table S2: Malignant disease spectrum.

## Data Availability

The data that support the findings of this study are available from the corresponding authors upon reasonable request.
